# Investigation on the Potential Correlation Between *TP53* and Esophageal Cancer

**DOI:** 10.3389/fcell.2021.730337

**Published:** 2021-10-27

**Authors:** Lihua Yao, Xiaowu Zhong, Guangcheng Huang, Qiang Ma, Lei Xu, Hong Xiao, Xiaolan Guo

**Affiliations:** ^1^Department of Clinical Laboratory, Affiliated Hospital of North Sichuan Medical College, Nanchong, China; ^2^Department of Laboratory Medicine, North Sichuan Medical College, Nanchong, China

**Keywords:** esophageal cancer, expression, prognosis, *TP53*, bioinformatics

## Abstract

**Background:** *TP53* family members play an indispensable role in various human cancers, while the gene expression profiles, prognostic value, and potential mechanism in esophageal cancer (ESCA) are yet unclear.

**Methods:** The expression and roles of *TP53* family members in ESCA were investigated using the Cancer Genome Atlas (TCGA), Tumor Immune Estimation Resource (TIMER), Kaplan–Meier plotter, gene set enrichment analysis (GSEA), and UALCAN databases. The expression of *TP53* between ESCA and the corresponding adjacent tissues was validated using qRT-PCR. Furthermore, the effects of *TP53* on esophageal squamous cell carcinoma (ESCC) cell migration and proliferation were examined using the Transwell assay, scratch test, and crystal violet assay. The correlation between *TP53* and mTOR pathways was evaluated by Western blotting.

**Results:** This study showed a correlation between high mRNA expression of *TP53* members (*TP53*, *TP63*, and *TP73*) and clinical cancer stages and nodal metastasis status in ESCA patients. Moreover, the expression of *TP53* was significantly associated with the overall survival (OS) of ESCA patients. Additional experiments verified that the mRNA of *TP53* was upregulated in ESCC patients. Moreover, the downregulated expression of *TP53* significantly retarded ESCC cell migration and proliferation and might activate the mTOR signaling pathway and inhibit *TP53*-dependent autophagy.

**Conclusion:** *TP53* has a prognostic value in ESCA and may be a leading factor in promoting ESCA pathogenesis.

## Introduction

Esophageal cancer (ESCA) is the leading cause of digestive system cancer mortality (Global Burden of Disease Cancer Collaboration et al., 2015). About 300,000 people die of esophageal cancer worldwide, with about 460,000 new cases every year (Ferlay et al., 2013). It is histologically defined by both esophageal squamous cell carcinoma (ESCC) and esophageal adenocarcinoma (EAC). Approximately 90% of ESCA cases worldwide are ESCC (Arnold et al., 2015) and the major histological type is in East Asian countries like China (Lin et al., 2013), while the morbidity and mortality of ESCC are ranked fifth and fourth, respectively (Chen W. et al., 2015). The etiology of ESCA is associated with ethnicity, genetics, and dietary habit (Abnet et al., 2018). It is characterized by epigenetic abnormalities and disorders in the signaling pathways, which is consistent with other cancers (Chen J. et al., 2015). The 5-year overall survival (OS) rate of ESCA patients after the operation has greatly improved over the last couple of decades, while the OS and the prognosis are still poor, and approximately 20% are due to late diagnosis (Dubecz et al., 2012). The ESCA morbidity concealment, rapid progression, and lack of effective means for the early diagnosis are the main causes of high mortality (Chen et al., 2007). Thus, identifying specific and sensitive biomarkers and understanding the pathogenetic mechanisms underlying ESCA are valuable for clinicians to choose appropriate treatments to improve the survival rate of patients.

The *TP53* family of transcription factors, including *TP53, TP63*, and *TP73*, plays key roles in biological and pathological processes of cancer and neural development (Agostini et al., 2018). These three proteins have a very similar domain organization and, hence, overlapping functions. These proteins also have unique functions: *TP53* regulates the stress response to suppress tumors (Levine, 2020b), *TP63* is essential for ectoderm development (Santos-Pereira et al., 2019), and *TP73* regulates both stress response and development (Levrero et al., 2000). *TP53* is a tumor suppressor gene associated with neoplastic disease (Bykov et al., 2018), while *TP53* gene mutations occur in about half of human cancers (Kandoth et al., 2013). Approximately 90% of ESCAs harbor *TP53* mutations (Gao et al., 2014), indicating that *TP53* has a significant role in ESCA. The primary function of *TP53* is as a transcription factor that protects the cells from various stresses, including autophagy, apoptosis, and senescence (Lane, 1992; Vousden and Prives, 2009; Ryan, 2011; Bieging et al., 2014). *TP53* gene mutations cause a loss of the tumor suppressor gene and also contribute to tumorigenesis, such as increased genomic instability and cell proliferation, enhanced invasion and metastasis, drug resistance, and inhibition of apoptosis (Oren and Rotter, 2010). However, only a few studies have assessed the correlation between *TP53* family members and ESCA. Therefore, a comprehensive analysis of the role of TP53 family in ESCA was imperative.

In the current study, we examined the mRNA expression of *TP53* family members on ESCA using TIMER and UALACN database. Next, we analyzed the correlation between clinicopathological features and prognostic values of *TP53* family members in ESCA. Additionally, we verified *TP53* mRNA expression and clinicopathological characteristics in ESCC tissue. Subsequently, downregulated *TP53* significantly inhibits ESCC cell proliferation and migration via mTOR signaling pathway and *TP53*-dependent autophagy. This study showed the prognostic value and potential biological function of *TP53* family members in ESCA, and hence, could be considered a diagnostic biomarker and a promising therapeutic target in ESCC.

## Materials and Methods

### Expression Analysis of *TP53* Family Members

Tumor Immune Estimation Resource (TIMER) is a public database that uses microarrays for estimating the gene expression. UALCAN is a cancer information analysis platform consisting of a transcriptional expression database, and the corresponding clinical information is based on the TCGA cancer data. In this study, the transcriptional expression data of TP53 family genes were obtained from the TIMER and UALCAN databases.

### Clinicopathological Analysis of *TP53* Family Members in Esophageal Cancer

The correlation between the expression of *TP53* family genes and clinicopathological parameters, including cancer stage, lymph node metastasis, tumor grade, and tumor histology was analyzed using the UALCAN database.

### Immune Infiltration Analysis

Furthermore, the immune infiltration of *TP53* family members in ESCA was evaluated using TIMER. The scatterplots of *TP53* family members were generated to explore the correlation between the gene expression level and the abundance of immune cell infiltration using Spearman’s correlation analysis.

### Survival Analysis

In this study, we used the Kaplan–Meier plot to analyze the prognostic value of mRNA expression of *TP53* family members in ESCA. Patients with ESCC and EAC were categorized into high- and low-expression groups, according to the median values of mRNA expression.

### Populations and Ethics Statement

This study was approved by the Medical Ethics Committee of the Affiliated Hospital of North Sichuan Medical College; the hospital is located in Nanchong, Sichuan Province, China. The patients provided their written informed consent to participate in this study. Between July 2015 and March 2016, cancer tissues and paracarcinoma normal tissues samples were obtained from 65 patients with ESCC who had undergone esophagectomy at the Department of Cardiothoracic Surgery, without preoperative chemotherapy or radiation. All patients were pathologically diagnosed with ESCC using surgical specimens and biopsies. All the tissue samples were surgically isolated within 30 min to excise an appropriate amount of tumor and normal tissue, soaked in RNAlater solution (Ambion, Carlsbad, CA, United States), and stored at −80°C until further processing.

### Cell Culture

Human normal esophageal epithelial cell line (HET-1A) was purchased from American Type Culture Collection (ATCC, Manassas, VA, United States), and human ESCC cell lines (TE1 and Kyse150) were obtained from the Cell Bank of Shanghai Institute of Cell Biology (Chinese Academy of Medical Sciences, Shanghai, China). All the cells were maintained in RPMI-1640 medium (Gibco, Grand Island, NY, United States) containing 10% fetal bovine serum (FBS, Gibco) and 1% penicillin-streptomycin (Invitrogen, Waltham, MA, United States) at 37°C with 5% CO_2_ in the humidified incubator.

### RNA Extraction and Reverse Transcription

The total RNA was extracted from ESCC tissues and cell lines using the TRIzol reagent (Ambion) according to the manufacturer’s instructions. Reverse transcription was performed using the Transcriptor First Strand cDNA Synthesis Kit (Roche Diagnostics, Indianapolis, IN, United States), and subsequently, the cDNA was collected and stored at −80°C.

### Quantitative Real-Time PCR

Quantitative Real-Time PCR was detected by the Lightcycler 480 Real-time PCR system (Roche, Mannheim, Germany) under the following conditions: 95°C for 5 min, followed by 40 cycles of 10 s at 95°C, 15 s at 62°C, 72°C for 10 s, 65°C for 60 s, and 37°C for 30 s. For *TP53* mRNA detection, forward primer 5′-CCAGGGCAGCTACGGTTTC-3′ and reverse primer 5′-CTCCGTCATGTGCTGTGACTG-3′. β-Actin was used as an internal control: forward primer 5′-GGACTTCGAGCAAGAGATGG-3′ and reverse primer 5′-AGCACTGTGTTGGCGTACAG-3′. Each experiment was performed in triplicate, and the relative expression of mRNA was normalized to the endogenous expression level of β-actin through the 2^–Δ^ ^Δ^ ^Ct^ method.

### Plasmid and Stable Cell Transfection

Kyse150 and TE1 cells were selected for further functional research. Plasmids encoding knockdown *TP53* (sh-*TP53*) and corresponding controls (sh-NC) were purchased from Hanbio (Hanbio, Shanghai, China). According to the manufacturer’s protocol, 50–60% of confluent ESCC cells were transfected with plasmids using Lipofectamine 2000 reagent (Invitrogen, Waltham, MA, United States). The stably transfected cells were screened in the presence of 2 μg/ml puromycin for 14 days (Sigma, Chicago, IL, United States).

### Western Blot

Total protein was extracted from tissues and cell pellet lysis, and the concentration of protein in the lysate was determined using the Pierce BCA Protein Assay Kit (Thermo Fisher Scientific, Rockford, IL, United States). Proteins were separated by 12% sodium dodecyl sulfate polyacrylamide gel electrophoresis (SDS-PAGE) and transferred to polyvinylidene difluoride (PVDF) membranes (Millipore; Merck KGaA, Darmstadt, Germany). After blocking with 5% skimmed milk for 1 h, the membranes were probed with primary antibodies at 4°C overnight: anti-*TP53* (1:1000) and LC3 (1:500) were purchased from Cell Signaling Technology (CST; Danvers, MA, United States), while anti-p-AKT^Ser473^ (1:500), anti-p-mTOR (1:500), anti-p-P70S6K, anti-p-4EBP1, and P62 (1:500) were purchased from Sigma (St. Louis, MO, United States), and anti-GAPDH (1:5000) was purchased from CST, which served as a loading control. Then, the membrane was incubated with a secondary antibody for 2 h. The protein expression was visualized using enhanced chemiluminescence (ECL) reagent (Thermo Fisher Scientific), and the concentration of proteins was quantified by Image J software.

### Cell Proliferation Assay

Cell proliferation was analyzed using the crystal violet assay. Briefly, 5 × 10^5^ stably transfected cells were seeded into each well of a six-well plate in duplicates and cultured for 1–4 days. Then, the cells were fixed with pre-cooled 10% formaldehyde for 20 min and stained with 0.1% crystal violet for 15 min. The reaction was stopped by 10% glacial acetic acid, and the absorbance (450 nm) was measured on a SpectraMax Paradigm microplate reader (Molecular Devices, Sunnyvale, CA, United States).

### Cell Migration Test

For the Transwell migration assay, 3 × 10^5^ cells suspended in medium without serum were added to the upper chamber of the Transwell plates (Corning Inc., Corning, NY, United States), while 20% FBS-medium was added to the lower chamber. After incubation for 24 h, the cells in the upper chamber were removed by a cotton swab, while the migrated cells attached to the bottom were fixed with 10% formaldehyde and stained with 0.1% crystal violet. Images were captures using a camera equipped with a BX41 Olympus microscope (Leica, Wetzlar, Germany). Three independent experiments were performed.

### Gene Set Enrichment Analysis of *TP53* in Esophageal Cancer

Gene set enrichment analysis is an analysis method for genome-wide expression of the microarray data that compares the genes with predefined gene sets. Herein, we used the GSEA approach to analyze the biological functions of *TP53* gene with 81 ESCC tissues obtained from TCGA using the ClusterProfiler and ggplot2 package in the R software. Using | NES| > 1 and *p*-value < 0.05 as the threshold of GSEA, pathways were considered significantly enriched when they fulfilled the sub-conditions.

### Statistical Analysis

The two sets of data of cancer tissues and adjacent tissue mRNA expression are skewness distribution, as analyzed by the non-parametric statistical method Wilcoxon test. The difference between *TP53* expression and clinicopathologic characteristics was analyzed by Fisher’s exact test. The data in this study were analyzed by GraphPad Prism 7.0 software (GraphPad, San Diego CA, United States) and the R software. *p* < 0.05 indicates a statistically significant difference.

## Results

### High Transcriptional Expression of *TP53* Family Members in Esophageal Cancer Patients

**FIGURE 1:**
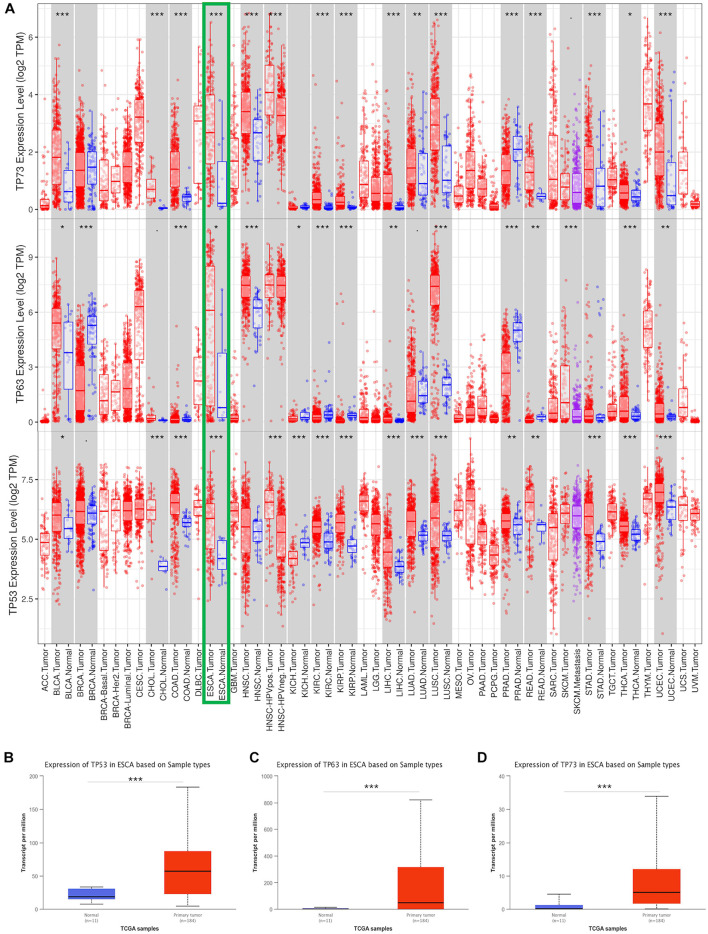
Transcriptional expression of *TP53* family member analysis. The mRNA levels of *TP53* family members in 33 types of cancers from the TIMER database **(A)**. The expression of *TP53* family members between ESCA and non-cancerous tissues from the TCGA **(B–D)**. * indicates *p* < 0.05, ** indicates *p* < 0.01, *** indicates *p* < 0.001.

To explore the transcriptional expression of *TP53* family members in ESCA, we analyzed 33 types of cancers tissues and the corresponding normal tissues using the TIMER database. As shown in Figure 1A, the mRNA expression of *TP53*, *TP63*, and *TP73* was significantly upregulated in ESCA tissues compared to normal tissues. Moreover, the comparison of the expression of *TP53* family members based on the TCGA database showed a significantly higher expression of all *TP53* members in ESCA tissues than normal specimens (Figures 1B–D).

### Association of Transcriptional Expression of *TP53* Family Members and the Clinical Parameters in Esophageal Cancer Patients

**FIGURE 2:**
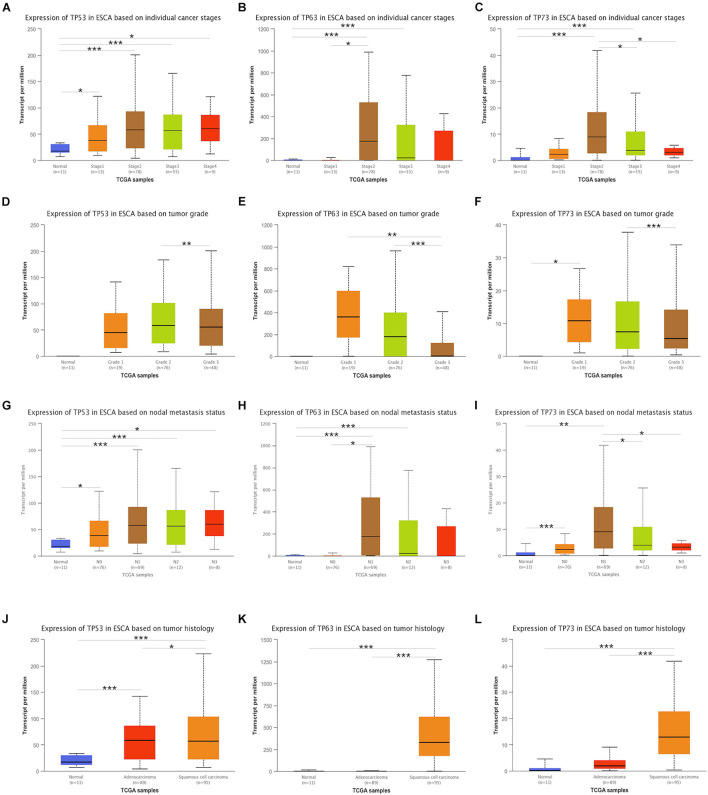
Association between transcriptional expressions of *TP53* family members and clinical parameters. The transcriptional expressions of *TP53* family members associated with cancer stages, tumor grade, nodal metastasis status, and tumor histology **(A–L)**. * indicates *p* < 0.05, ** indicates *p* < 0.01, and *** indicates *p* < 0.001.

The investigation of the association between clinicopathological features and mRNA expression of the *TP53* family by UALCAN showed that the mRNA expressions of *TP53* family members were significantly and positively associated with cancer stages, tumor grade, node metastasis, and tumor histology. Compared to non-cancerous tissues, the upregulated expression of *TP53* family members was correlated with stage I, II, III, and IV (Figures 2A–C), while no statistically significant difference was detected in *TP63* and *TP73* mRNA expression between stages II and IV compared to non-cancerous tissues. However, the expression of TP53 family members only between tumor grade 2 and grade 3 had obvious statistic difference. and TP73 mRNA expression in tumor grade 1 was significantly higher than non-cancerous tissues (Figures 2D–F).

Next, the association of the expression of *TP53* family members to the node metastasis of ESCA patients was analyzed. As shown in Figure 2G, the mRNA expression of *TP53* was positively associated with the node metastasis of ESCA patients. However, the expression of *TP63* and *TP73* did not differ significantly with nodal metastasis status in N0, N3 and N2, and N3, respectively (Figures 2H,I).

We also analyzed the association between mRNA expressions of *TP53* family members and the tumor histology of ESCA patients. As shown in Figures 2J–L, the mRNA expression of *TP53* family members was significantly upregulated in ESCC tissues compared to normal tissues, while that of *TP63* and *TP73* did not show any remarkable difference in ECA tissues. Taken together, the mRNA expression of *TP53* was significantly associated with the clinicopathological parameters of ESCA patients.

### Immune Infiltration in Correlation With Expression of *TP53* Family Members in Esophageal Cancer Patients

**FIGURE 3:**
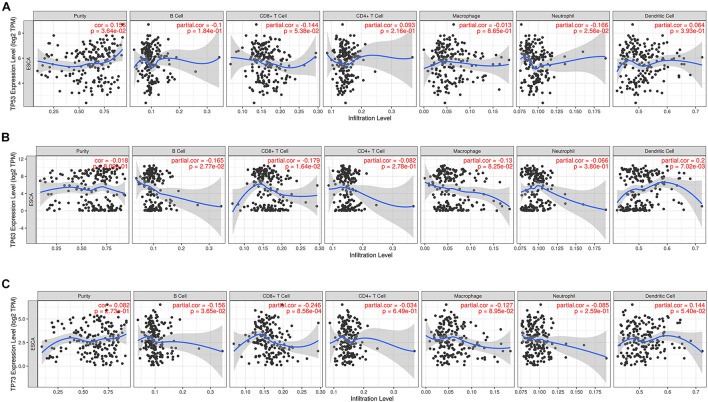
The correlation between *TP53* family members and immune infiltration cells. The expression level of *TP53* family members was associated with immune cell infiltration **(A–C)**.

The correlation between the mRNA expression of *TP53* family members with immune infiltration levels in ESCA was investigated using the TIMER database. The mRNA expression of *TP53* was obviously related to tumor purity and neutrophils as shown in Figure 3A. However, B cells, CD8 + T cells, and dendritic cells were weakly not significantly correlated with the mRNA expression of *TP63* (Figure 3B). On the other hand, the mRNA expression of *TP73* showed a remarkable correlation with infiltrating levels of B cell and CD8 + T cells in ESCA (Figure 3C). The above data illustrated that *TP53* family members were remarkably related to the infiltration of immune cells, indicating significant effects of *TP53* family members on ESCA.

### Prognostic Value of *TP53* Family Members in Esophageal Cancer Patients

**FIGURE 4:**
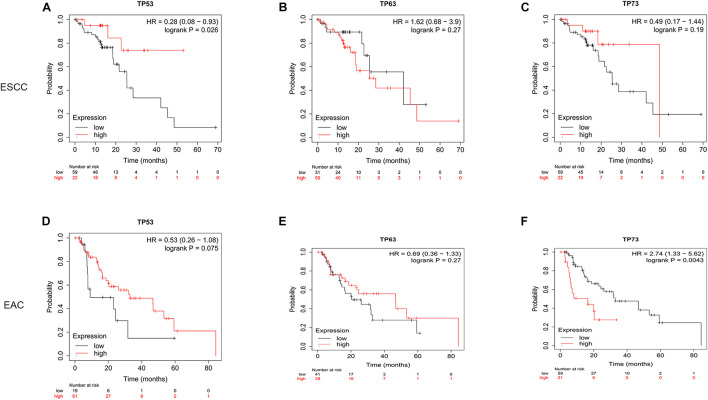
Prognostic value of *TP53* family members in ESCA patients. Overall survival curves for *TP53* family members in ESCA patients **(A–F)**.

The prognostic value of *TP53* family members in ESCA patients was analyzed by Kaplan–Meier plotter. As shown in Figure 4, *TP53* [hazard ratio (HR) = 0.28 and log-rank *p* = 0.026] showed a significantly good prognosis in ESCC patients when mRNA expression was upregulated, and *TP73* (HR = 2.74 and log-rank *p* = 0.0043) showed a negative correlation between high mRNA expression and significantly positive OS in EAC patients. However, the mRNA expression of *TP63* did not show any statistically significant association with the prognosis of both ESCC and EAC patients. These results might provide additional evidence about the prognostic biomarkers for ESCA. Thus, we focused on the role of the *TP53* in subsequent experiments.

### Verification mRNA Expression of *TP53* in Esophageal Squamous Cell Carcinoma Patients

**FIGURE 5:**
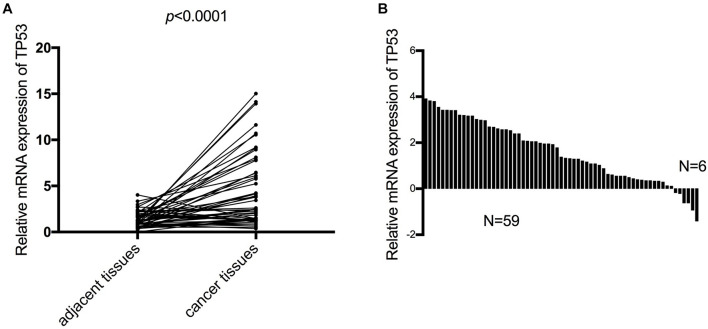
The mRNA expression of *TP53* validated in 65 patients with ESCC. The mRNA expression of *TP53* and the matched adjacent tissues detected by qRT-PCR. Three independent experiments were conducted **(A)**. High mRNA expression of TP53 more than 90% (59/65) of ESCC tissues compared with adjacent normal tissues **(B)**.

Furthermore, we verified the mRNA expression of *TP53* in 65 ESCC patients by RT-qPCR. The results of expression in ESCC patient tissues and the corresponding adjacent tissues were consistent with the analysis using the TCGA database, wherein the mRNA expression of *TP53* was upregulated in ESCC tissues compared to the paired adjacent normal tissues (Figure 5A). Notably, >90% (59/65) of ESCC tissues expressed high levels of TP53, while only 10% (6/65) of matched normal tissues expressed high levels of *TP53* (Figure 5B). These findings strongly validated that the mRNA expression of *TP53* is upregulated in ESCC.

The association between mRNA expression of *TP53* and the clinical factors in 65 ESCC patients was analyzed. As shown in Table 1, high mRNA expression of *TP53* in ESCC was positively correlated with TNM stage (*p* = 0.007) and lymph node metastasis (*p* = 0.030) in ESCC patients, while no association was observed with other clinicopathological features, including age, gender, and differentiation. Conversely, the correlation between the expression of *TP53* and TNM stage and lymph node metastasis was consistent with the analysis using the TCGA database.

**TABLE 1:** Association of clinicopathological data and expression of *TP53* in ESCC patients.

**Patient characteristics**	**TP53 expression**		**χ^2^**	***P-*value**
	**High (*n* = 59)**	**Low (*n* = 6)**		
Age			0.437	0.508
<65	28	2		
≥65	31	4		
Gender			0.271	0.603
Male	45	4		
Female	14	2		
Tumor size			0.538	0.764
<3 cm	15	2		
3–5 cm	40	4		
>5 cm	4	0		
Tumor location			1.06	0.589
Upper	9	1		
Middle	40	3		
lower	10	2		
TNM stage			12.2	0.007*
I	7	4		
II	31	2		
III	19	0		
IV	2	0		
Lymph node metastasis			4.697	0.030*
Yes	27	0		
No	32	6		
Differention			1.036	0.596
Well	20	1		
Moderate	27	4		
Poor	12	1		

Using a Fisher’s exact test. The *p*-value was set at 0.05 and * indicates *p* < 0.05.

### *TP53* Regulates the Proliferation and Migration of Esophageal Squamous Cell Carcinoma Cells

**FIGURE 6:**
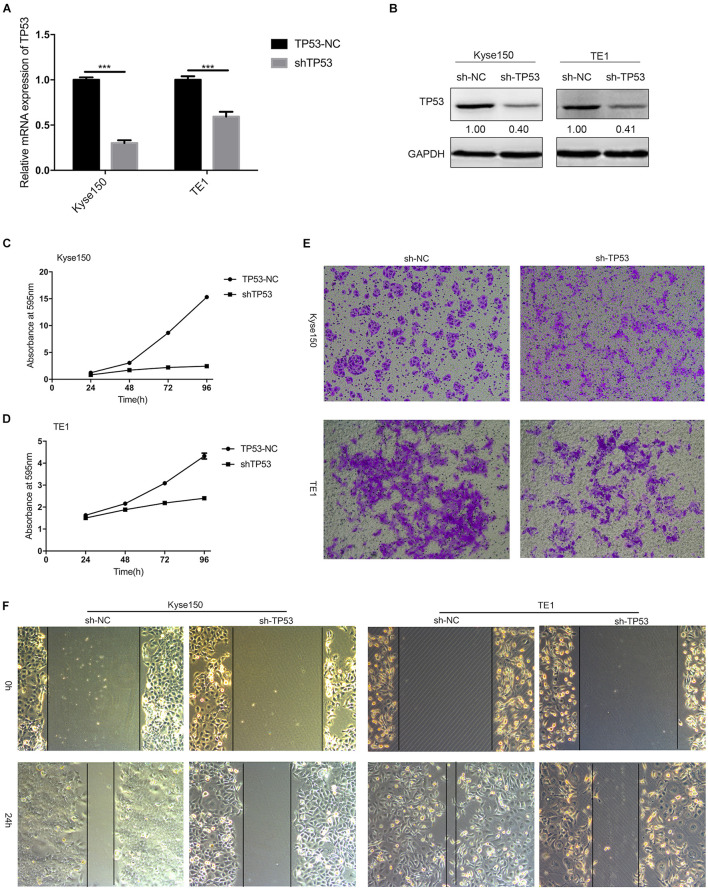
*TP53* regulates the proliferation and migration of ESCC cells. KYSE-150 and TE1 cells were stably transfected with plasmids (sh-NC and sh-*TP53*). The mRNA expression and protein expression of *TP53* were determined by qRT-PCR and Western blotting **(A,B)**. Crystal violet assay demonstrated that knockdown of *TP53* significantly inhibited cell proliferation **(C,D)**. Transwell and Scratch test showed downregulated expression of *TP53* decreased cell migration **(E,F)**. Data are presented as mean ± SD; *** indicates *p* < 0.001.

To further investigate the potential biological function of *TP53*, the expression of *TP53* in Kyse150 and TE1 ESCC cells was knocked down by stable transfection with sh-*TP53* plasmids, while sh-NC plasmid was transfected in ESCC cells as a control. As shown in Figures 6A,B, the mRNA expression and the protein level of *TP53* were downregulated by sh-*TP53* in ESCC cells, which in turn significantly reduced the growth rate of Kyse150 and TE1 cells, as assessed by cell proliferation assay (Figures 6C,D). The Transwell assay showed that the downregulated expression of *TP53* suppressed the migratory ability of ESCC cells (Figure 6E); the wound healing assay also displayed a similar trend (Figure 6F). Together, these findings indicated that *TP53* is involved in the regulation of proliferation and migration of ESCC cells.

### *TP53* Regulates mTOR Signaling Pathway in Esophageal Squamous Cell Carcinoma Cells

**FIGURE 7:**
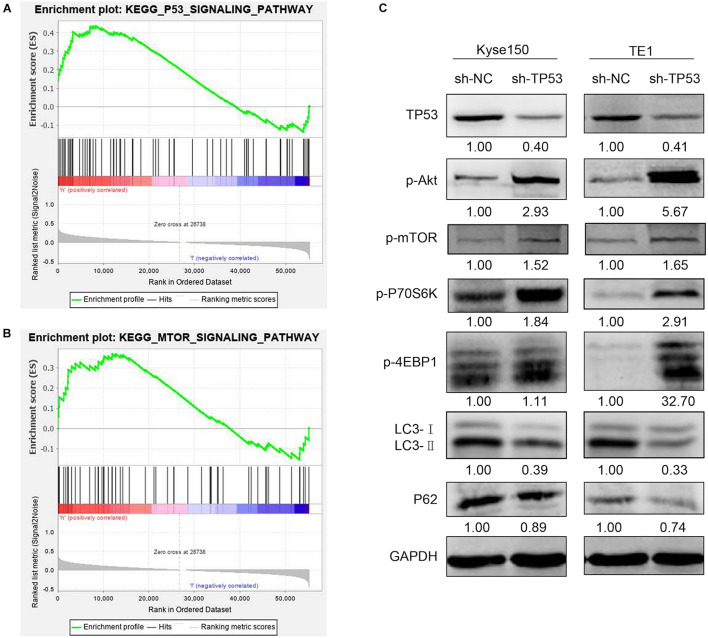
*TP53* regulated mTOR signaling pathway in ESCC cells. GSEA enrichment plots demonstrated that *TP53* was positively correlated with the *TP53* signaling pathway and mTOR signaling pathway **(A,B)**. Western blotting analysis of the mTOR signaling pathway- and autophagy-associated proteins **(C)**.

The above results indicated that *TP53* gene plays a vital role in tumorigenesis of ESCC. GSEA was utilized to describe the *TP53* gene on the cellular process to investigate the underlying biological function. As shown in Supplementary Table 1, 27 gene sets were significantly enriched; among these, *TP53* signaling pathway, mTOR signaling pathway, NOTCH signaling pathway, the mitogen-activated protein kinase (MAPK) signaling pathway, and pathway in cancer cell cycle are closely related to tumorigenesis. The enriched gene sets of *TP53* and AKT-mTOR were shown by normalized enrichment score value ranking in Figures 7A,B. These findings indicated that *TP53* promotes ESCC development by regulating AKT-mTOR signaling pathway. The investigation of AKT-mTOR signaling pathway by Western blotting in stably transfected sh-*TP53* ESCC cells elucidated the molecular mechanisms underlying *TP53* in tumorigenesis and development of ESCC. As shown in Figure 7C, the expression of *TP53* was significantly decreased when transfected with sh-*TP53* cells compared to sh-NC cells. The phosphorylation of p-p70S6K and p-4EBP1 was increased in *TP53*-downregulated ESCC cells, indicating that the downregulation of *TP53* significantly enhanced the activation of AKT-mTOR pathway. Also, P62 and LC3-II also showed a declining trend compared to the sh-*NC* group. Collectively, these results suggested that the downregulation of *TP53* activates the transduction pathway of AKT-mTOR and inhibits autophagy, which may be responsible for its tumor-suppressive function.

## Discussion

Various molecules are known to be associated with the development and progression of ESCA. *TP53* family members are known as tumor suppressors participate in the development of several tumors. The *TP53* family members and their interactions with other factors promoted progression of thyroid cancer (Manzella et al., 2017). The correlation between the *TP53* family members and the Notch signaling pathway is often inactivated in the cutaneous squamous cell carcinoma (Missero and Antonini, 2014). All *TP53* family members are abnormally expressed in bladder cancer, and *TP73* overexpression is associated with aggressive tumor phenotype (Papadogianni et al., 2014). The immunostaining in oral squamous cell carcinomas revealed that the *TP53* family members are biomarkers that could contribute to the diagnosis and monitoring of high-risk precancerous lesions of the oral epithelium (Bidaud et al., 2010). The abnormal expression of *TP63* and *TP73* is associated with hematological malignancy grade and poor prognosis (Alexandrova and Moll, 2012). *TP53* family members constitute an interacting network involved in cellular responses to chemotherapeutic drugs in gastrointestinal cancer (Vilgelm et al., 2010). Moreover, a differential expression of *TP53* family members is observed in the antimitotic agent vincristine-treated TP*53*-deficient breast cancer cells (Vayssade et al., 2002). However, the function and prognosis of *TP53* family members in ESCA are yet unknown. To the best of our knowledge, our study is the first clarifying the systematic analysis of *TP53* family genes in ESCA patients using multiple bioinformatics databases. The results showed that mRNA expression of *TP53*, *TP63*, and *TP73* in ESCA tissues was significantly higher than in normal tissues. Moreover, the expression of TP53 was upregulated in both ESCC and EAC, while high expression of *TP63* and *TP73* was observed only in EAC. Furthermore, we investigated the association between the clinicopathological data and the expression of *TP53* family members of ESCA patients. The mRNA expression of *TP53* was remarkably correlated with cancer stages (stage I, II, III, and IV) and nodal metastasis status in ESCA tissues, while the expression of *TP63* and *TP73* was correlated with cancer stages II and III. The study also displayed the lack of correlation between mRNA expression of the *TP53* family and tumor grade.

To address the expression and function of *TP53* in ESCA, we detected the level of *TP53* in ESCA tissue. Owing to the high morbidity rate of ESCC in China, we selected ESCC cell lines (Kyse150 and TE1) for further study. *TP53* is a tumor-suppressor gene but is mutated in about 50% of cancers, thereby regulating the proliferation of various tumor cells (Egashira et al., 2011). A high expression of *TP53* has been observed in many malignancies (Hinds et al., 1990; Iggo et al., 1990; Samuels-Lev et al., 2001), including esophageal cancer (Huang et al., 2014; Yao et al., 2014; Xie et al., 2017). Several studies found that the expression of *TP53* in cancer tissues of ESCC patients could be utilized to analyze the survival and prognosis of esophageal cancer (Yao et al., 2014; Xie et al., 2017; Melling et al., 2019). These findings strongly suggested that *TP53* is involved in the tumorigenesis of ESCA. Herein, we also verified that the mRNA expression of *TP53* was remarkably upregulated in ESCC tissues. Also, the analysis of clinicopathological parameters showed that TP53 levels were positively correlated with TNM stage and lymph node metastasis. In addition, Kaplan–Meier analysis revealed that overexpression of *TP53* in ESCC patients is correlated with prognosis. The downregulated expression of *TP53* significantly suppressed the ESCC cell growth and migration. These findings revealed that *TP53* exerts an oncogenic role in the initiation and progression of ESCC.

Previous studies have also demonstrated the role of *TP53* in immune response and tumor microenvironment. Blagih and Levine reported that *TP53* regulates immune cells to participate in B-cell and CD8 killer T-cell response of cancer cells (Blagih et al., 2020; Levine, 2020a). Vadakekolathu et al. (2020) found that *TP53* abnormalities are involved in immune response and beneficial for immunotherapy of acute myeloid leukemia. Based on the TCGA-ESCA dataset, we found that *TP53* was significantly correlated with the abundance of immune cells. Our results might imply that *TP53* may carry out an immune escape role in the ESCA microenvironment.

In order to further explore the molecular mechanism of *TP53* regulating the proliferation and migration in ESCC cells, we analyzed the effects of *TP53* gene on the cellular process using the GSEA approach. The results suggested that *TP53* and mTOR signaling pathways promote ESCC progression by influencing the pathways in cancer. Subsequently, we demonstrated that the activation of AKT-mTOR pathway was significantly enhanced in *TP53* downregulated ESCC cells. In terms of molecular mechanisms, *TP53* regulates cell cycle and apoptosis and protects the cells from DNA-damaging agents (Case and Domann, 2014; de Assis and Isoldi, 2014). In addition, *TP53* is also involved in other functions, such as immune response, senescence, cellular differentiation, angiogenesis, DNA metabolism, and senescence (Suzuki and Matsubara, 2011). Additionally, *TP53* mediates autophagy in some pathological factors. Feng et al. (2005) found that the induction of *TP53* by DNA damaging agents inhibited mTOR and induced autophagy in mouse embryo fibroblasts. Budanov and Karin (2008) also demonstrated that *Sestrin1* and *Sestrin2* are the products of two *TP53* target genes, which inhibit mTOR via binding and activating AMPK. Importantly, loss of *Sestrin2* significantly reduced *TP53*-mediated autophagy. The combined data indicated that *TP53* inhibits the mTOR pathway and subsequently induces autophagy. The mTOR pathway plays a vital role in the regulation of cell growth and proliferation (Hay and Sonenberg, 2004). In mammals, mTOR is regulated by PI3K, PI3K-dependent kinase 1, and AKT. Subsequently, mTOR regulates cellular processes by regulating its downstream targets including p70S6 kinase and eIF4E binding protein 1 (4EBP1), which are critical regulators of translation (Brown et al., 1995; Brunn et al., 1997). In this study, the phosphorylation of AKT, mTOR, 70S6K, and 4EBP1 was increased in *TP53*-downregulated ESCC cells. Furthermore, P62 and LC3-II showed a declining trend in *TP53*-knockdown cells. These findings indicated that the downregulation of *TP53* expression mediates cell translation via mTOR pathway. Moreover, *TP53* downexpression activates mTOR and inhibits autophagy. Thus, it could be speculated that *TP53* mediates the mTOR pathway to regulate the proliferation and autophagy in ESCC cells. However, these findings need to be validated clinically.

## Conclusion

This study showed that *TP53* is upregulated in ESCC tissues and plays a vital role in ESCC cell proliferation and migration. It also might activate the mTOR signaling pathway and inhibit TP53-dependent autophagy. Thus, this might provide new insights into the potential role of *TP53* as a diagnostic biomarker and valuable therapeutic target in ESCC.

## Data Availability Statement

The datasets presented in this study can be found in online repositories. The names of the repository/repositories and accession number(s) can be found in the article/Supplementary Material.

## Ethics Statement

The studies involving human participants were reviewed and approved by the Medical Ethics Committee of the Affiliated Hospital of North Sichuan Medical College. The patients/participants provided their written informed consent to participate in this study.

## Author Contributions

XG, LY, and XZ conceived and designed the experiments. LY and XZ performed the experiments and wrote the initial draft of the manuscript. GH and QM contributed to the statistical analysis. GH contributed to the clinical data collection. LX and HX were involved in the provision of study materials. XG reviewed and edited the manuscript. All authors reviewed and approved the final manuscript.

## Conflict of Interest

The authors declare that the research was conducted in the absence of any commercial or financial relationships that could be construed as a potential conflict of interest.

## Publisher’s Note

All claims expressed in this article are solely those of the authors and do not necessarily represent those of their affiliated organizations, or those of the publisher, the editors and the reviewers. Any product that may be evaluated in this article, or claim that may be made by its manufacturer, is not guaranteed or endorsed by the publisher.

## Supplementary Material

Supplementary Table 1 GSEA of pathways significantly represented in ESCA.
